# Extracellular Vesicle-Enclosed Oxidative Stress- and Inflammation-Related microRNAs as Potential Biomarkers of Vitamin D Responsivity: A Pilot Study on Inflammatory Bowel Disease Patients with or without COVID-19

**DOI:** 10.3390/antiox13091047

**Published:** 2024-08-28

**Authors:** Giorgia Ammirata, Maddalena Arigoni, Danilo Licastro, Gian Paolo Caviglia, Michela Disabato, Ghania Zubair, Cristina Bezzio, Simone Saibeni, Amedeo De Nicolò, Jessica Cusato, Alice Palermiti, Alessandra Manca, Emanuela Tolosano, Stefano Cozzini, Marcello Mancini, Fiorella Altruda, Antonio D’Avolio, Davide Giuseppe Ribaldone, Ugo Ala, Sharmila Fagoonee

**Affiliations:** 1 Department of Molecular Biotechnology and Health Sciences, Molecular Biotechnology Centre “Guido Tarone”, University of Turin, 10126 Turin, Italy; giorgia.ammirata@unito.it (G.A.); maddalena.arigoni@unito.it (M.A.); emanuela.tolosano@unito.it (E.T.); fiorella.altruda@unito.it (F.A.); 2 AREA Science Park, Padriciano, 34149 Trieste, Italy; danilo.licastro@areasciencepark.it (D.L.); stefano.cozzini@areasciencepark.it (S.C.); 3 Gastroenterology Unit, Department of Medical Sciences, University of Turin, 10126 Turin, Italy; gianpaolo.caviglia@unito.it (G.P.C.); micheladisabato94@gmail.com (M.D.); davrib_1998@yahoo.com (D.G.R.); 4 Department of Mathematics “Giuseppe Peano”, University of Turin, 10126 Turin, Italy; ghania.zubair@unito.it; 5 IBD Centre, IRCCS Humanitas Research Hospital, 20089 Rozzano, Italy; cristina.bezzio@hunimed.eu; 6 Department of Biomedical Sciences, Humanitas University, 20072 Pieve Emanuele, Italy; 7 Gastroenterology Unit, Rho Hospital, ASST Rhodense, 20017 Milan, Italy; saibo@tiscali.it; 8 Laboratory of Clinical Pharmacology and Pharmacogenetics, Department of Medical Sciences, University of Turin, 10149 Turin, Italy; amedeo.denicolo@unito.it (A.D.N.); jessica.cusato@unito.it (J.C.); alice.palermiti@unito.it (A.P.); alessandra.manca@unito.it (A.M.); antonio.davolio@unito.it (A.D.); 9 Institute for Biostructure and Bioimaging, CNR, Via T. De Amicis 95, 80145 Naples, Italy; marcello.mancini@ibb.cnr.it; 10 Department of Veterinary Sciences, University of Turin, Grugliasco, 10095 Turin, Italy; 11 Institute for Biostructure and Bioimaging, CNR, Molecular Biotechnology Centre “Guido Tarone”, 10126 Turin, Italy

**Keywords:** vitamin D, inflammatory bowel diseases, SARS-CoV-2, COVID-19, extracellular vesicles, oxidative stress, inflammation, biomarkers, bioinformatics, Crohn’s disease, ulcerative colitis

## Abstract

The relationship between serum 25-hydroxyvitamin D (25(OH)D) levels, genomic response to vitamin D (Vit.D), and positivity to SARS-CoV-2 remains understudied. In this pilot study, during the follow-up of patients with Inflammatory Bowel Disease (IBD) and COVID-19, we investigated this issue by analyzing the molecular contents of serum extracellular vesicles (EVs) from six groups of IBD patients (n = 32), classified according to anti-SARS-CoV-2 status, 25(OH)D level, and Vit.D supplementation, by small RNA-seq. This analysis revealed differentially expressed miRNAs, PIWI-RNA, transfer RNA, small nucleolar RNAs, and protein-coding RNAs in the EVs obtained from these cohorts of IBD patients. Experimental validation evidenced a statistically significant increase in miR30d-5p, miR150-5p, Let-7f-5p, and Let-7a-5p in the anti-SARS-CoV-2-positive and low 25(OH)D and Vit.D supplemented groups with respect to the non-Vit.D supplemented group, indicating their responsiveness to Vit.D treatment. Bioinformatics analysis highlighted the regulation of these validated miRNAs by oxidative stress and inflammation, hallmarks of IBD and COVID-19. Our study reports an unprecedented panel of circulating EV-enclosed inflammation- and oxidative stress-related miRNAs, the potentiality of which, as biomarkers for Vit.D responsivity in IBD patients, needs to be explored in future studies on larger cohorts in order to allow clinicians to optimize current treatment strategies upon viral infection.

## 1. Introduction

Vitamin D (Vit.D) is an essential nutrient and pre-hormone that regulates a broad spectrum of physiological activities. The biologically active form of Vit.D (1,25-dihydroxyvitamin D [1,25(OH)_2_D]) can modulate the functional activities of both innate and adaptive immune cells, which express the Vit.D receptor (VDR), hence influencing gene expression with genome- and transcriptome-wide impacts and regulating immune response, inflammation, oxidative stress, and gut microbiota profile [1,2,3,4,5]. Recent epidemiological data have revealed that Vit.D deficiency is associated with higher morbidity rates in multiple infectious diseases, autoimmune disorders, and various components of the metabolic syndrome, as well as in cancer, pointing out the anti-tumorigenic effects of this vitamin [6,7]. A low serum level of 25-hydroxyvitamin D (25(OH)D), which indicates the body’s Vit.D status, was found to be correlated with the morbidity of several upper respiratory tract infections, such as flu, and lately, Coronavirus disease 2019 (COVID-19) [8]. Regarding COVID-19, several observational studies have reported an inverse correlation between serum Vit.D levels and the incidence and severity of COVID-19 [9]. We have recently demonstrated that a Vit.D level over 30 ng/mL was associated with SARS-CoV-2 infection devoid of symptoms in patients with Inflammatory Bowel Disease (IBD) [10]. This uncertainty has also led COVID-19 patients to undergo self-medication with Vit.D resulting in hypercalcemia [11]. Importantly, COVID-19 can promote oxidative stress through several pathways, including TNFα and NFκB, which can worsen IBD symptoms [12,13,14]. IBD encompasses a range of inflammatory conditions that affect the digestive system and are marked by chronic episodes of relapse and remission. IBD comprises two main forms—Crohn’s disease (CD) and ulcerative colitis (UC), the etiology of which remains elusive. A crosstalk among oxidative stress, gut microbiota, and immune response has been recently proposed [15]. Typically, the onset and progression of IBD coincide with enduring oxidative stress and an imbalance in the generation of Reactive Oxygen Species (ROS), alongside inflammatory reactions triggered by gut microbiota dysbiosis [15]. Fine-tuning the level of cellular ROS is important, as physiological levels of ROS can serve as second messengers to modulate normal cell functions such as immune defense, whereby intracellular hydrogen peroxide can trigger the migration of leukocytes to the sites of injury in several ways, for example [16]. On the other hand, overproduction of ROS can cause damage to cellular biomolecules (DNA, proteins, and lipids), gradually leading to cellular senescence and death [17]. Importantly, Vit.D deficiency is common in IBD patients, and Vit.D levels could predict disease activity as well as response to biological therapy, which are higher in IBD patients [18]. A recent meta-analysis revealed that Vit.D supplementation can dampen the risk of clinical relapse in IBD patients, specifically in UC patients [19]. Thus, IBD patients should be monitored for Vit.D levels and supplemented on a registered dietitian-prescribed diet as per recent indications in order to establish the doses and supplementation modality [20,21]. However, the relationship between serum 25(OH)D levels, the genomic response to Vitamin D (Vit.D), and the positivity to SARS-CoV-2 in the context of IBD remains unclear.

Extracellular vesicles (EVs) have emerged as promising biomarker vehicles. EVs are a heterogeneous group of nano-sized particles enclosing bioactive molecules, such as nucleic acids, proteins, lipids, and metabolites, within a bilayer membrane [22]. EVs are released in all body fluids, and RNA encapsulated within EVs has become a focal point in biomarker research due to its “message in a bottle” type of active release into the bloodstream under pathological conditions [22]. Importantly, EV-enclosed RNA molecules exhibit markedly greater resistance to RNAse activity compared to their unbound, cell-free counterparts. This enhanced stability has paved the way for the integration of various RNA types into the development of a more refined and effective biomarker panel for diagnostic as well as therapeutic purposes. Several factors influence the release and content of EVs. In this regard, oxidative stress and inflammation occurring in IBD are major contributors [17]. EVs have been shown to carry biomolecules indicative of the pathological condition, such as in the case of cholestasis-induced liver fibrosis, or IBD, at the preclinical level [23,24]. Clinical studies have been limited to small cohorts of patients. For example, it was shown that EV-closed membrane proteins and interleukin-6 could help in classifying COVID-19 patients according to the severity (mild, moderate, and severe) of the disease [25]. Moreover, bronchoalveolar lavage fluid EVs from sarcoidosis patients were significantly enriched in Vit.D–binding protein with respect to those of controls and could potentially serve as a sarcoidosis diagnosis biomarker [26]. Vit.D-binding protein was also reduced in plasma microglia-derived EVs from major depressive disorder patients compared to those from matched healthy controls [27]. Thus, in the present study, we performed an unprecedented “omics”-based analysis to investigate whether serum EV miRNA contents could prove an important source of biomolecules for the evaluation of Vit.D responsiveness in IBD patients differentially infected with SARS-CoV-2, as the IBD course is particularly susceptible to Vit.D levels and patients are at high risk of developing Vit.D insufficiency and deficiency [28]. To our knowledge, this is the first report on EV-miRNAs as potential biomarkers for Vit.D responsiveness in IBD patients.

## 2. Materials and Methods

### 2.1. Patient Selection, Vit.D Supplementation, Blood Sampling, and Quantification of 25(OH)D Levels

The criteria for IBD patient enrollment, clinical parameters, and SARS-CoV-2 serological data have been previously described [10]. Briefly, following blood sampling, plasma was prepared for 25(OH)D dosage by ultra-ultra-high-performance liquid chromatography coupled with tandem mass spectrometry (UHPLC-MS/MS) [10]. Patients with Vit.D deficiency (serum 25(OH)D < 30 nmol/L) were treated with cholecalciferol at 25.000 UI once/month if they were not under Vit.D therapy, or with 25.000 UI twice/month or 50.000 UI once/month if they were already under treatment with cholecalciferol 25.000 UI once/month (Dibase^TM^, ABIOGEN PHARMA, Pisa, Italy). This study was approved by the Ethics Committee of Città della Salute e della Scienza Hospital, Turin, Italy (protocol number 0109499, 12 November 2020). Serum was prepared by centrifuging the clotted blood at 2000× *g* for 15 min at 4 °C. The supernatant was stored at −80 °C until further processing.

### 2.2. EV Enrichment and Characterization

Circulating EVs were prepared from serum, as previously described [23]. Briefly, serum was thawed on ice and Exoquick™ (System Biosciences, LLC, Palo Alto, CA, USA) added according to the manufacturer’s instructions. Pelleted EVs were resuspended in PBS and visualized on a NanoSight LM10 instrument (Particle Characterization Laboratories, Novato, CA, USA). The size profile and concentration of the particles in plasma samples were evaluated with NTA 3.2 software.

Proteins from EVs were extracted using RIPA buffer (Sigma-Aldrich, Milano, Italy), followed by quantification using the Bradford method (Bio-Rad, Milano, Italy), according to the manufacturer’s instructions. Ten micrograms of protein were analyzed by Western blotting for the determination of the presence of exosomal markers (anti-CD81 and anti-HSP90 antibodies, System Biosciences, LLC, Palo Alto, CA, USA) in EVs as previously described [23].

### 2.3. EV RNA Extraction and miRNA Sequencing

For the extraction of total RNA, EV pellets were reconstituted in 200 μL of PBS, and 1 mL of QIAzol Lysis Reagent (Qiagen, Milan, Italy) was added. RNA was isolated using the miRNeasy Serum/Plasma Kit (Qiagen) with the QIAcube instrument (Qiagen, Milan, Italy), ensuring consistent results by eliminating operator-dependent variability. The total RNA concentration was measured using the Qubit RNA HS kit (Thermo Fisher Scientific, Milan, Italy), and samples were stored at −80 °C for future use. The RNA was quantified using the NanoDrop 2000 spectrophotometer (Thermo Fisher Scientific, Milan, Italy).

MiRNA sequencing was conducted by Area Science Park (Trieste, Italy) using Illumina sequencing technology. MiRNA-Seq libraries were prepared with the QIAseq miRNA Library Kit (QIAGEN, Hilden, Germany) and sequenced on the NovaSeq 6000 platform (Illumina, San Diego, CA, USA) in 2 × 100 paired-end mode. MiRNA identification in the samples was carried out using QIAseq miRNA-NGS data analysis software, utilizing Single Read for read type and Read 1 Cycles 75 as read cycles [29]. The QIAseq miRNA sequencing FASTQ files were processed in the following steps: (1) Calibrate miRBase entries. Manual calibration was created for miRNA entries with identical or nearly identical sequences in the miRBase mature database, resulting in the formation of a new combined miRNA entry for each specific miRNA set. (2) Trim adapter and low-quality bases. The reads were processed using Cutadapt (cutadapt.readthedocs.io/en/stable/guide.html, accessed on 25 October 2022) to trim off the 3′ adapter and remove low-quality bases. (3) Identify insert and unique molecular index (UMI) sequences. After trimming, the insert sequences and UMI sequences were identified. Reads with fewer than 16 base pair (bp) insert sequences (too_short_reads) or fewer than 10 bp UMI sequences (UMI_defective_reads) were discarded. (4) Align insert sequences. A sequential alignment strategy was employed to map to various databases (perfect match to miRBase mature, miRBase hairpin, noncoding RNA, mRNA, and otherRNA, and finally a second mapping to miRBase mature with up to two mismatches allowed) using Bowtie (bowtie-bio.sourceforge.net/index.shtml, accessed on 25 October 2022). Only unmapped sequences advanced to the next step at each stage. Read counts for each RNA category (miRBase mature, miRBase hairpin, piRNA, tRNA, rRNA, mRNA, and otherRNA) were calculated based on the mapping results (miRNA_Reads, hairpin_Reads, piRNA_Reads, etc.). MiRBase V21 was utilized for miRNA, and piRNABank was used for piRNA.

### 2.4. Bioinformatics Analysis

Differential expression analysis of the different classes of transcripts (miRNA, PIWI-interacting RNA, transfer RNA, small nucleolar RNAs, and protein-coding RNAs) was conducted through the edgeR (release 4.2.0) library in the R environment (release 4.4.0) and Rstudio (Build 524). Transcripts were filtered out by filterByExpr (with default parameters), and their expression was normalized by the Trimmed Mean of M-values (TMM) method. Differentially expressed transcripts were selected, imposing a cut-off of 0.58 in absolute value for the logFC and a *p*-value < 0.01. Expression data for differential expression analysis are based on counts (number of reads); for samples sequenced more than once, the reference value used for transcripts is the average of counts among different sequencings of the same sample.

TF-miRNA regulation relationships were investigated through TransmiR version 2.0 [30]. Evidence of possible interactions between experimental data and high-throughput technologies is reported.

### 2.5. Real-Time PCR Analysis

For miRNA expression analysis, RNA was extracted from serum EVs (independent serum samples) as described above and analyzed using miRCURY Locked Nucleic Acid (LNA) miRNA PCR panels following the manufacturer’s instructions (Qiagen, Milano, Italy). Internal housekeeping miRNAs were chosen from RNA-seq data (miR-486-5p, -92a-3p, -122-5p, -23a-3p, and -22-3p) (Figure S1). As comparable results were obtained by qRT-PCR using two chosen unmodulated miRNAs, miR22-3p and miR122-5p, all successive normalizations were performed using miR122-5p.

### 2.6. Statistical Methods

Continuous variables were presented as medians with their interquartile ranges (IQR). Categorical variables were described in terms of counts and percentages. For comparing continuous variables, the Mann-Whitney test was used for independent samples, while the Wilcoxon test was applied to paired samples. All correlation-related statistical analyses were conducted using MedCalc^®^ version 20.104 (MedCalc Software Ltd., Ostend, Belgium), with a *p*-value of ≤0.05 considered indicative of statistical significance. 

For comparing multiple experimental groups, a one-way analysis of variance (ANOVA) followed by Bonferroni’s *post hoc* test was performed using GraphPad Prism5.0 software. * *p* < 0.05, ** *p* < 0.01, *** *p* < 0.001, and **** *p* < 0.0001 were considered statistically significant. A Student’s *t*-test was also used to compare groups C versus E or D versus F, with a *p*-value < 0.05 or <0.01 considered statistically significant.

## 3. Results

### 3.1. IBD Patients and Clinical Data, Vit.D Concentration and Supplementation, and COVID-19

Thirty-two IBD patients were selected for this study, and the patients’ characteristics are described in Table 1.

IBD Patients were further divided into 4 groups (groups A–D; Table 2), according to their SARS-CoV-2 serological results and Vit.D status.

**Figure 1:**
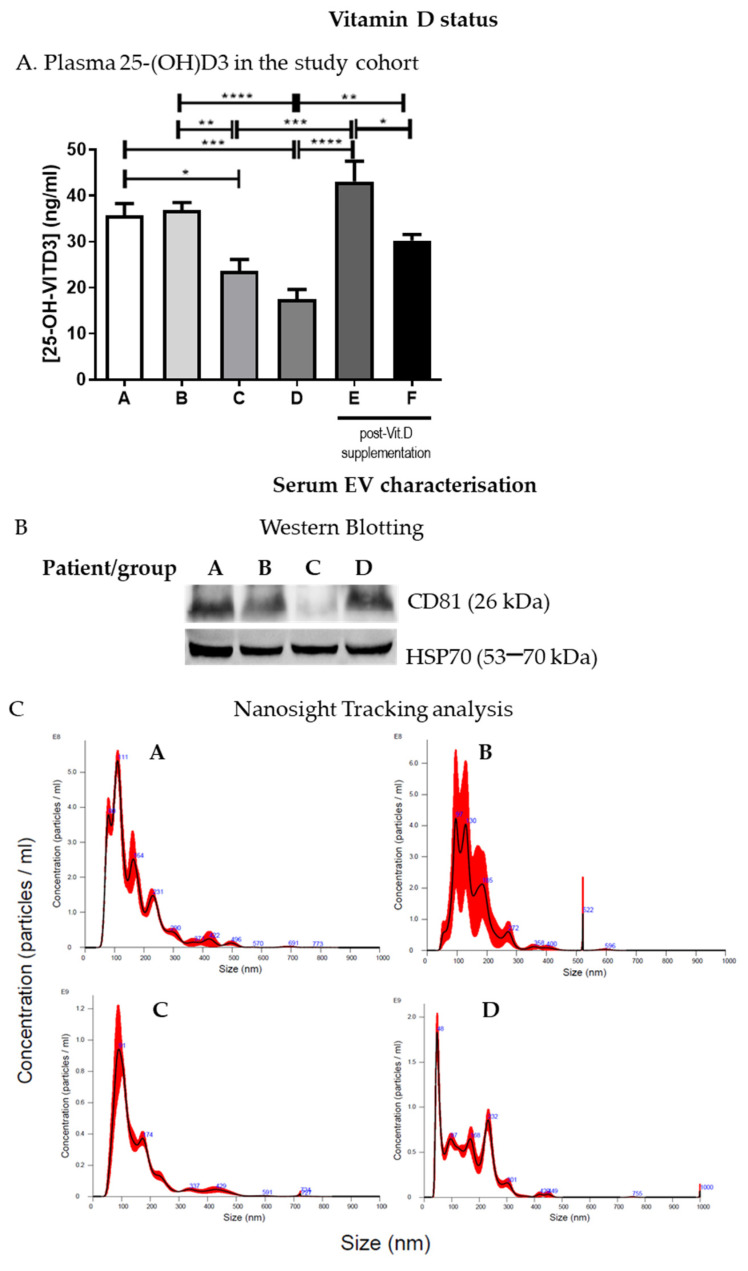
IBD patients 25(OH)D levels and serum EV characterization. (**A**). Plasma 25(OH)D levels are shown for the study cohort divided into groups (A, n = 4; B, n = 8; C, n = 7; D, n = 8; E, n = 4; F, n = 5). (**B**). Serum EV characterization by Western blotting; a representative blot is shown. (**C**). Nanosight tracking analysis of serum EVs; representative graphs are shown. * *p* < 0.05, ** *p* < 0.01, *** *p* < 0.001, and **** *p* < 0.0001.

IBD patients in groups A and B had >30 ng/mL 25(OH)D levels with respect to those of groups C and D (<30 ng/mL). Thus, patients with <30 ng/mL 25(OH)D (groups C and D) underwent Vit.D supplementation for 1 month as described in the methods section, and the level of plasma 25(OH)D was assessed thereafter. Plasma 25(OH)D basal levels and SARS-CoV-2 positivity or negativity are reported in Figure 1 and Table 2, respectively. As expected, following Vit.D supplementation, there was a statistically significant increase in 25(OH)D levels in these patients (groups E and F) (Figure 1A).

### 3.2. IBD Patients’ Serum EV Enclose miRNAs Indicative of Oxidative Stress- and Inflammation-Related Pathways

The cargoes of EVs extracted from plasma and serum have been considered potential biomarkers for numerous conditions, including liver injury induced by alcohol and drugs [31,32]. EVs were enriched from IBD patients’ serum and characterized by Western blotting (Figure 1B and Figure S2) and Nanosight tracking analysis as previously described [23]. EV size and concentration in the samples from the different groups varied from 101.5 +/− 50.8 nm to 171.3 +/− 2.9 nm and from 4.19 × 10^10^ +/− 2.16 × 10^10^ particles/mL to 1.54 × 10^11^ +/− 5.37 × 10^9^ particles/mL, respectively (Figure 1C), but no statistically significant differences were observed in the number or concentration of EVs among the groups (A–D).

Small RNA-seq of serum EVs derived from the 6 groups of IBD patients (A–F) revealed the presence of different species of RNA (Figure S3). In particular, mRNA (Figure S3A and Table S1), miRNA (Figure S3B and Table S2), piRNA (Figure S3C and Table S3), tRNA (Figure S3D and Table S4), or other tRNA (Table S5) were particularly enriched in the EVs derived from patients belonging to the different groups.

**Figure 2:**
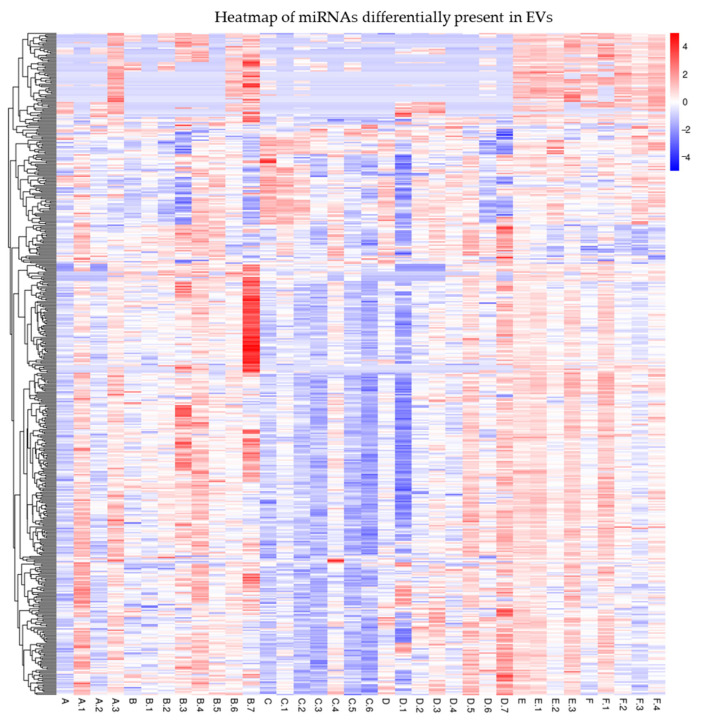
Heatmap of miRNAs differentially expressed among EVs of the different groups of IBD patients under study. Table S6 shows mapping_Samples_ID.

Interestingly, a further analysis of the serum EV-enclosed miRNAs revealed by small RNA-seq showed several differentially expressed miRNAs (Figure 2). We chose to validate several miRNAs (Table 3) by qRT-PCR according to the following criteria: Reads > 1000; FC > 2 in at least two groups of patients; relevance to the Vit.D, inflammation, or IBD pathway. 

**Figure 3:**
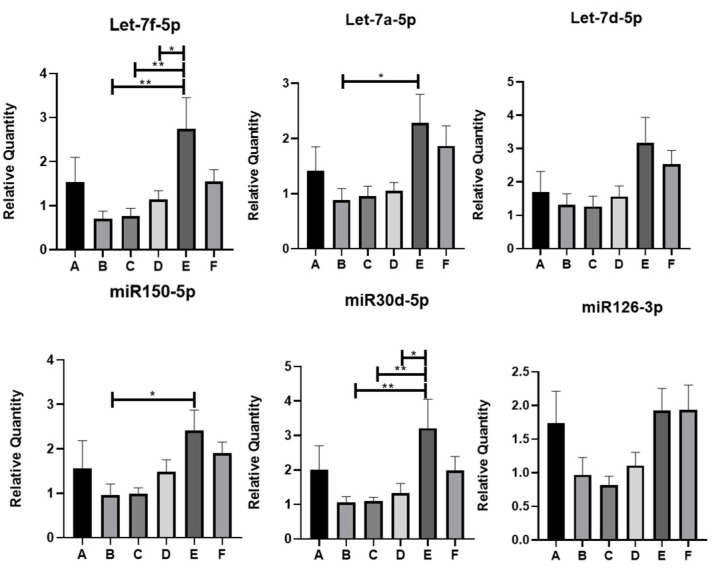
QRT-PCR analysis of miRNAs differentially expressed in the EVs of IBD patients. * *p* < 0.05; ** *p* < 0.01. miR122-5p was selected as internal housekeeping miRNA. A-F are the groups of IBD patients.

Using qRT-PCR, the following miRNAs were found to be statistically significantly different at least between two groups of IBD patients under study: Let-7f-5p, Let-7a-5p, miR150-5p, and miR30d-5p (Figure 3). In particular, Let-7f-5p and miR30d-5p showed a statistically significant increase in group E (SARS-CoV-2 IgG positive, Vit.D < 30 ng/mL, and Vit.D supplemented) with respect to groups B (SARS-CoV-2 IgG negative, Vit.D > 30 ng/mL), C (SARS-CoV-2 IgG positive, Vit.D < 30 ng/mL), and D (SARS-CoV-2 IgG negative, Vit.D < 30 ng/mL). Let-7a-5p and miR150-5p levels also enhanced expression in group E with respect to other groups of patients; however, statistical significance was reached only between groups E and B. On the other hand, the trends in expression levels of the other miRNAs evidenced by miRNome analysis could be confirmed by qRT-PCR analysis among some groups, but these did not reach statistical significance (Figure 3 and Figure S4).

In order to get insights into the mechanisms by which the four validated miRNAs were regulated in the context of IBD and SARS-CoV-2 infection, we bioinformatically interrogated the TransmiR v2.0 database to find potential transcription factor (TF) binding sites on the promoter of these miRNAs [30]. As oxidative stress and inflammation are hallmarks of IBD and SARS-CoV-2 infection, we specially searched for binding sites for TFs related to these pathways. Interestingly, this analysis pointed out the presence of binding sites for several oxidative stress and inflammation-related TFs on the validated miRNAs (Table S8).

### 3.3. Serum-Derived EV miRNA Expression Indicates Vitamin D Responsivity in IBD Patients Positive for SARS-CoV-2 IgG

IBD patients who were positive for SARS-CoV-2 IgG responded significantly better to Vit.D supplementation with respect to the negative ones (Figure 1A). Interestingly, the qRT-PCR-validated miRNAs Let-7a-5p, Let-7f-5p, miR150-5p, and miR30d-5p were upregulated in the circulating EVs of these IBD patients with respect to those who were not infected by SARS-CoV-2 (Table 4).

### 3.4. miRNA Expression in IBD Patients’ Serum-Derived EVs and COVID-19 Severity

We analyzed EV-miRNA expression according to the presence of different specific symptoms associated with COVID-19. Among validated EV-miRNAs, we did not observe any difference between patients with asymptomatic COVID-19 as compared to those that experienced at least one symptom. Furthermore, no differences were observed according to the following symptoms: fever, cough, diarrhea, vomit, headache, and anosmia/ageusia. Conversely, we observed a significant lower expression of miR126-3p, miR191-5p, miR23a-3p, miR30d-5p, Let7a-5p, and Let7d-5p in patients who experienced dyspnea as compared to those who did not (*p* < 0.05).

## 4. Discussion

In the present study, we addressed the question of whether, in patients with IBD and normal levels of Vit.D who have developed antibodies against SARS-CoV-2, a milder COVID-19 symptomatology has occurred with respect to those with low levels of Vit.D. Patients with IBD usually have low levels of Vit.D and often receive standardized supplementation therapy. Vit.D supplementation, however, as a treatment for diseases remains controversial, partly due to conflicting clinical study results. Moreover, it has become evident that a “one-size-fits-all” approach is not appropriate as not all individuals, due to genetic and epigenetic factors, respond uniformly to Vit.D. Single-nucleotide polymorphisms in Vit.D-related genes, for example, may affect the bioavailability of 25(OH)D, highlighting the need to identify biomarkers that can indicate responsiveness to Vit.D rather than solely relying on measuring the level of the major circulating form of Vit.D, 25(OH)D [33,34]. This necessity is underscored by the global prevalence of Vit.D deficiency (serum 25(OH)D < 30 nmol/L) and insufficiency (serum 25(OH)D < 50 nmol/L) at 15.7% and 47.9%, respectively [35]. 

A few studies have tackled the problem of identifying molecular responses to Vit.D supplementation. Changes in expression of the primary Vit.D target genes are promising biomarkers for the evaluation of the responsiveness of the Vit.D signaling system in order to optimize current treatment strategies in a personalized way. In this regard, Carlberg et al. reported the genes *CD14* and *THBD* as promising biomarkers capable of reflecting the transcriptomic response of human tissues to Vit.D supplementation [36]. As early as 24 h post-1,25(OH)_2_D stimulation, there was an induction in the expression of VDR target genes in peripheral blood mononuclear cells (PBMCs), which represent a useful source of biomarkers [37]. A panel of 12 differentially expressed VDR target genes was found between Vit.D-supplemented and control PBMCs. Of these, only the expression of *CD38* and *TMEM37* correlated with serum 25(OH)D levels, hence allowing us to distinguish between low and high responders. A follow-up of 5 months upon Vit.D supplementation indicated that the vitamin most probably induces epigenetic changes in the selected VDR target genes rather than transcriptomic ones [37]. Clinical studies have been carried out to classify patients according to their responsiveness to Vit.D: NCT01479933 and NCT02063334 (ClinicalTrials.gov) [38]. The ligand-dependent binding of VDR to a multitude of genomic sites, along with the epigenome-wide impact of Vit.D, has also been documented [2,38] However, implementing this in clinical practice poses significant challenges.

A limited number of studies have been performed to investigate the serum levels of Vit.D and circulating miRNA expression profiles. In Vit. D intervention trials, notable differences were observed between the treatment groups and between baseline and follow-up measurements [39]. For instance, Jorde et al. analyzed the plasma miRNA profile, at baseline and at study end, of 159 obese male patients included in a 12-month intervention study with Vit.D supplementation [40]. Out of the 136 miRNAs detected in 10 subjects in 2 pilot studies, 12 miRNAs showed significant differences between baseline and 12-month plasma samples. These miRNAs were subsequently analyzed in a cohort of patients supplemented with Vit.D for 12 months versus those who received placebo. While one miRNA, miR221, showed a statistically significant difference between baseline and the 12-month’s placebo group, only one miRNA, miR532-3p, showed a significantly positive correlation with serum Vit.D levels at baseline. No changes in selected miRNA profiles were observed after treatment with Vit.D for 12 months with respect to baseline, showing the difficulty in demonstrating the effect of Vit.D on the miRNA plasma profile [40]. MiRNA expression profiles also changed in IBD patients according to their Vit.D status. Atanassova et al. recently reported, in an abstract presented at the 17th Congress of European Crohn’s and Colitis Organization, that the levels of miRNA-28_1 and miRNA-1228-3p_1 are increased in IBD patients who normalized their 25(OH)D levels, concluding that there is a correlation between the serum expression of different miRNAs and the Vit.D levels in patients with IBD (P079, https://www.ecco-ibd.eu/images/2_Congresses_Events/2022/4_Abstracts/MASTER_ECCO22_Abstract_Book.pdf, last visited on the 8 March 2024). The following miRNAs exhibit alterations in expression during IBD: miRNA-21, miRNA-122a, miRNA-155, and miRNA-150, all of which are associated with intestinal epithelial permeability; moreover, miRNA-126, miRNA-146a, and miRNA-155 are connected to the regulation of both innate and adaptive immune responses in intestinal inflammation [41]. 

To our knowledge, no studies have been performed on the EV-encapsulated miRNAs in IBD patients in association with their Vit.D status or their response to viral infection. Through small RNA-seq profiling of serum EV RNA, we identified an array of differentially modulated miRNAs in IBD patients classified according to Vit.D status. None of these miRNAs, however, showed statistically significant changes according to SARS-CoV-2 positivity or COVID-19 symptoms, except for dyspnea, probably due to the small cohort of patients included in this pilot study. On the other hand, as a novelty, the characterization of EVs from patients with IBD with normal or low levels of Vit.D, led to the identification of four miRNAs capable of predicting the response of IBD patients following Vit.D administration.

As oxidative stress and inflammation are common hallmarks of IBD and COVID-19 pathogenesis, and considering the fact that low Vit.D levels in IBD patients may exacerbate oxidative stress, we assessed whether the miRNAs differentially present in the EVs of the study cohorts had oxidative stress- or inflammation-related transcription factor binding sites on their promoters [15,42,43]. Interestingly, MYC, which can influence ROS homeostasis through different processes such as proliferation, hypoxia, and mTORC1-mediated metabolism, could bind the promoter of all four validated miRNAs in this study [44]. Other oxidative stress-related transcription factors, such as HIF1A, CREB1, SP-1, and FOXO1, already reported to play a role in the oxidative stress pathway, have binding sites on the promoters of some of these miRNAs, indicating that these biomolecules can be released into circulating EVs following the oxidative stress inherent to IBD and COVID-19 [45]. In fact, it has been demonstrated that oxidative stress can modulate EV shedding as well as their molecular cargo, which can in turn affect redox status in recipient cells [46].

MiR150-5p and Let-7a-5p showed an increased expression trend in EVs in the Vit.D supplemented groups compared to those of the non-supplemented ones, reaching statistical significance only in the SARS-CoV-2 IgG-positive, Vit.D-treated cohort with respect to the SARS-CoV-2 IgG-negative, non-Vit.D-treated cohort of IBD patients, indicating that the rise in the levels of these two miRNAs was more likely to be caused by Vit.D supplementation and independently of viral infection. Interestingly, miR-150 has already been shown to be involved in IBD [47]. The ROS-responsive miR150-5p can be regulated by NFκB1 and FOXO1, which are transcription factors involved in oxidative stress and inflammation. Importantly, a link exists between miR150-5p and VDR. Li et al. demonstrated, using fibroblasts from ankylosing spondylitis patients’ ligament tissue from joint capsules, that overexpression of miR150-5p could inhibit osteogenic differentiation by decreasing VDR expression through the targeting of its 3′UTR [48]. This aspect has yet to be explored in the context of the chronic inflammation generated during the course of IBD. On the other hand, it was shown that circulating Let-7a/b levels are positively correlated with Vit.D intake depending on the VDR genotype (absence of *BsmI* restriction site) of elderly blood donors [49]. Vit.D. treatment can also regulate the expression of a number of miRNAs, including the let-7 family. For instance, VDR can bind the VDRE in the promoter of Let-7a-2 and promote its expression in lung cancer cells (A549) [50]. A previous study highlighted that TNFα regulates the expression of Let-7 miRNAs [51]. Importantly, in IBD, TNFα production is induced by ROS, and patients can be prescribed anti-TNFα therapies, which lead to a reduction in oxidative stress in this pathology [52]. The Let-7a-5p promoter contains binding sites for HIF1α, which regulates the expression of genes involved in key cellular processes, including inflammation and oxidative stress [53]. 

The most interesting data were obtained with miR30d-5p and Let-7f-5p, which were upregulated in the circulating EVs of IBD patients following Vit.D supplementation with respect to non-treated groups, independent of anti-SARS-CoV-2 IgG status. Mir30d-5p is an intestinal miRNA that is regulated in response to dietary lipids and can modulate the gastrointestinal microbiota [54,55]. MiR30d-5p was also revealed as one of the differentially expressed miRNAs that could influence the progression of tuberculosis in patients. Importantly, miR30d-5p, together with miR27a-3p, could inhibit VDR involved in the progression of tuberculosis [56]. MiR30d-5p was also found to be differentially expressed in the UC and CD patients’ mucosa versus healthy controls [57]. This miRNA binds to the 3′UTR of VDR mRNA to dampen its expression, thereby enhancing the production of proinflammatory cytokine secretion and M1 macrophage differentiation [56]. The MiR30d-5p promoter has binding sites for the inflammation- and oxidative stress-associated transcription factors CREB, SP1, and HIF1α. In the context of IBD, these transcription factors may stimulate the expression and/or loading of miR30d-5p onto EVs to stimulate intercellular communication and modulate inflammatory responses [58]. On the other hand, Let-7f-5p was identified as one of the significantly upregulated miRNAs in active UC compared to controls [57]. Importantly, it was found that the forkhead box P3 (Foxp3)-positive Treg cells may play a role in halting diseases such as IBD by utilizing EV-enclosed miRNAs (exosomes) such as Let-7d-5p to inhibit pathogenic T helper 1 cells [59]. Moreover, Let-7d-5p was part of the top 10 IBD-related miRNA-regulatory modules, as revealed by a bipartite clustering approach [60]. The induction of the expression of Let-7d-5p, which targets high mobility group AT-hook 2 (HMGA2), by lipoprotein exposure was shown to modulate cholesterol influx in macrophages and hence regulate pathogenic processes such as those leading to cancer [61]. The role of Let-7d-5p in Vit.D metabolism is not clear yet and warrants further investigation. The expression of these EV-enclosed miRNAs upon Vit.D supplementation in the IBD context points out an interplay among these players, and their enrichment in EVs, especially in the context of SARS-CoV-2 IgG positivity, may signify an enhanced anti-inflammatory and anti-oxidative stress action of the EV released upon Vit.D supplementation.

Interestingly, IBD and COVID-19, in terms of immune activation pattern and cytokine storm during the inflammatory response, can influence each other, but it was demonstrated that SARS-CoV-2 infection alone could not reactivate IBD [62,63]. For instance, the cytokine storms caused by SARS-CoV-2 infection can generate a huge quantity of IL-17, which is also upregulated in IBD patients. Oxidative stress, resulting from an imbalance between the heightened production of ROS and diminished antioxidant defenses following SARS-CoV-2 infection, may also aggravate IBD and COVID-19 severity [15,64]. The ability to modulate these pathways to prevent IBD aggravation upon COVID-19 using, for example, Vit.D. is an attractive solution and warrants further larger prospective clinical studies. Circulating EVs may represent powerful biomarker sources to monitor the response to Vit.D supplementation.

The limitations of this pilot study mainly include the small cohort of IBD patients, the unknown VDR genotype, and the lack of follow-up for patients who tested negative for SARS-CoV-2 IgGs to determine if they were actually infected with the virus and to assess the clinical outcomes following Vit.D supplementation. Not all miRNAs selected from the small RNA-seq-generated miRNA profile could be validated experimentally, probably because of the low amount of EV-enclosed miRNAs, which required a higher number of qRT-PCR cycles to detect subtle changes in miRNA expression.

## 5. Conclusions

The data generated in the present study may contribute to the development of a panel of biomarkers capable of predicting the responsivity of IBD patients to Vit.D supplementation, especially in an era where low Vit.D levels have been found to be associated with long COVID syndrome [65]. Using the biomolecular contents of EV to analyze Vit.D responsiveness is a novelty of this study. The EV-enclosed miRNAs miR30d-5p, miR150-5p, Let-7a-5p, and Let-7f-5p are increased in the Vit.D supplemented group versus the non-supplemented one, indicating that incremented 25(OH)D levels induced an increase in the validated miRNA levels in the circulating EVs of IBD patients with anti-SARS-CoV2 positivity and warrant further studies in a larger cohort of patients. This could lead to personalized Vit.D supplementation and the possibility of specifically recommending Vit. D supplementation for IBD patients, which may contribute favorably to the prevention and prognosis of infectious diseases, thus dampening the socioeconomic burden on the health care system. In addition, the ability to assess response to Vit.D will assist clinicians (by including Vit.D in protocols) in modulating the severity of COVID-19, thereby reducing hospital stays. The Vit.D-responsive patient will be able to fight the SARS-CoV-2 infection more effectively and rapidly.

## Figures and Tables

**Table 1:** Overall patients’ characteristics. CD: Crohn’s Disease; IBD-U: unclassified Inflammatory Bowel Disease; UC: Ulcerative colitis.

Variable	
Median age (range) (n = 31)	49.0 (34.0–55.0)
Sex (n = 31)	48.4% males; 51.6% females
Type of IBD (n = 31)	64.5% CD; 6.5% IBD-U; 29.0% UC
Pre-study Vitamin D supplementation (n = 31)	32.3% No; 67.7% Yes
SARS-CoV-2 positivity (n = 31)	54.8% No (n = 17); 45.2% Yes (n = 14)
Hospitalization (n = 14)	85.7% No; 14.3% Yes
Ventilation (n = 14)	100% No
Death (n = 14)	100% No
Pneumonia (n = 14)	92.9% No; 7.1% Yes

**Table 2:** IBD Patients’ characteristics per group (g). y.o.: years old; IQR: interquartile range.

*Group*	*A*	*B*	*C*	*D*	*E*	*F*
Gender [Male%-Female%]; n	[50%-50%]; 4	[50%-50%]; 8	[42.9%-57.1%]; 7	[37.5%-62.5%]; 8	[50%-50%]; 4	[40%-60%]; 5
Median age (y.o.; IQR)	56.5 (38.0–64.0)	42.5 (39.0–50.0)	48.5 (33.0–56.0)	49.0 (28.5–54.5)	48.0 (30.0–59.0)	48.5 (25.5–57.5)
COVID-19 anamnesis (yes/no)	yes	no	yes	no	yes	no
Pre-study Vit.D supplementation (15,000–25,000 U/2 weeks or per month); (n)	50% (n = 2)	87.5 (n = 7)	42.9% (n = 3)	87.5 (n = 7)	50% (n = 2)	80% (n = 4)
Vit.D supplementation during this study (yes/no)	no	no	no	no	yes	yes

**Table 3:** The differentially expressed EV-enclosed miRNAs chosen for further qRT-PCR validation—logFC, *p*-value, and the compared conditions in which they were found statistically significant—are reported. The complete analysis is shown in Table S7.

miRNA	logFC	*p* Value	Comparison
hsa-let-7a-5p	−1.05214423	0.007905004	A vs. C
hsa-let-7a-5p	−1.78215526	0.006151082	E vs. C
hsa-let-7d-5p	−1.22782698	0.003414526	A vs. C
hsa-let-7f-5p	1.262294456	0.009947947	C vs. D
hsa-let-7f-5p	−1.84545328	0.004319857	E vs. C
hsa-miR-126-3p	−1.76896854	0.007664076	E vs. C
hsa-miR-126-5p	−1.71591194	0.007685279	E vs. C
hsa-miR-146a-5p	1.472326209	0.009641655	C vs. D
hsa-miR-150-5p	1.093716916	0.009059818	C vs. D
hsa-miR-150-5p	−1.54629612	0.004547162	E vs. C
hsa-miR-191-5p	−1.87556395	0.009207518	E vs. C
hsa-miR-21-5p	−1.35376327	0.007451891	E vs. C
hsa-miR-223-3p	−2.63160072	0.001610881	E vs. C
hsa-miR-30d-5p	−1.81395948	0.008396924	E vs. C
hsa-miR-423-5p	−1.94752558	0.005348262	E vs. C
hsa-miR-486-3p	−2.42802251	0.009517606	E vs. C

**Table 4:** miRNA expression in EVs of patients with low Vit.D levels who underwent Vit.D supplementation. Relative expression levels of miRNAs obtained by qRT-PCR on representative patients classified according to SARS-CoV-2 IgG status are shown prior to and following Vit.D treatment. In red: downregulation of expression with respect to starting level of expression. P1, P2, and P3 are three patients who had low Vit.D levels and underwent Vit.D supplementation.

miRNA	Low Vit.D			Vit.D Supplemented		
	P1 (SARS-CoV-2+)	P2 (SARS-CoV-2+)	P3 (SARS-CoV-2−)	P1 (SARS-CoV-2+)	P2 (SARS-CoV-2+)	P3 (SARS-CoV-2−)
Let-7a-5p	1.65148345	0.967622724	0.511047209	**2.627095884**	**2.846824659**	**0.837721773**
Let-7f-5p	1.600454936	0.723943443	0.303769668	**3.508573233**	**3.54355914**	**0.876204496**
miR150-5p	1.645731544	1.614814116	1.11826679	**2.655071171**	**3.350935823**	**1.013703793**
miR30d-5p	1.405683671	1.186686379	0.5156357	**3.506820284**	**5.054646657**	**1.130965628**

## Data Availability

Data will be provided on request.

## Supplementary Materials

The following supporting information can be downloaded: https://www.mdpi.com/article/10.3390/antiox13091047/s1, Figure S1: Box plots of miRNAs with unchanged expression across cohorts, considered housekeeping miRNAs. Figure S2: Uncropped images of Western blots. Figure S3: Heatmaps of RNA species revealed, by small RNA-seq analysis, in the circulating EVs of IBD patients belonging to the 6 groups under study. Table S6 shows mapping_Samples_ID. Figure S4: Expression of other EV-enclosed miRNAs analyzed by qRT-PCR. Table S1: DE mRNA overlap among the different groups. Table S2: DE miRNA overlap among the different groups. Table S3: DE piRNA overlap among the different groups. Table S4: DE tRNA overlap among the different groups. Table S5: DE and other RNA overlap among the different groups. Table S6 shows mapping_Samples_ID. Table S7: The mean reads of differentially expressed EV-enclosed miRNAs per group as assessed by miRNA sequencing. Table S8: Associations between miRNAs and specific TFs as found in the TransmiR v2.0 database. Some information is provided: miRNA TSS, the binding site position, and the action type (Regulation, Regulation (feedback), Activation, Repression, Repression (feedback)) according to the experimental data or the literature. The source of this putative evidence is either the NCBI SRA identifier or the PMID identifier, if experimental or from literature, respectively. The evidence of the association is: literature, Level 1 (predicted) or Level 2 (supported by high-throughput experimental data), and the Tissue related to this evidence.
