# Supplementary figures and images for: Extracellular Vesicle-Enclosed Oxidative Stress- and Inflammation-Related microRNAs as Potential Biomarkers of Vitamin D Responsivity: A Pilot Study on Inflammatory Bowel Disease Patients with or without COVID-19
Source: Antioxidants (Basel). 2024 Aug 28;13(9):1047. doi: 10.3390/antiox13091047 (PMC11429492; doi:10.3390/antiox13091047)

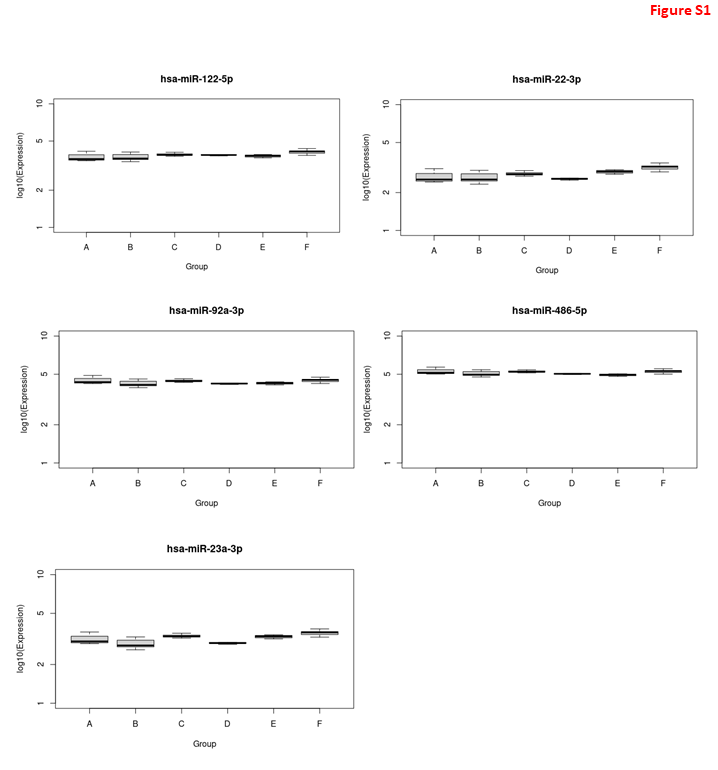

Supplement: Supplementary file 1 [file antioxidants-13-01047-s001.zip › Supplementary/Figure S1.tif]

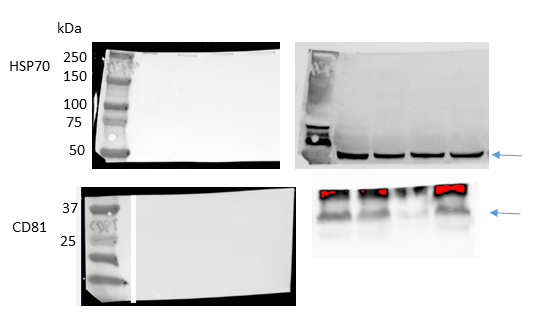

Supplement: Supplementary file 1 [file antioxidants-13-01047-s001.zip › Supplementary/Figure S2.TIF]

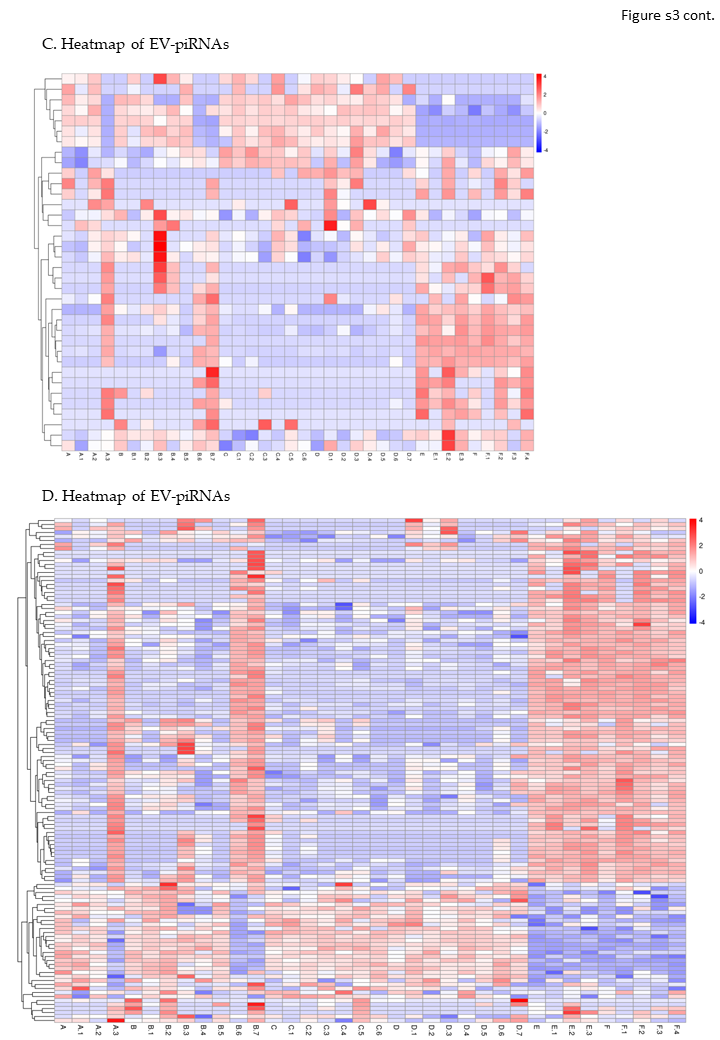

Supplement: Supplementary file 1 [file antioxidants-13-01047-s001.zip › Supplementary/Figure S3 cont.d.TIF]

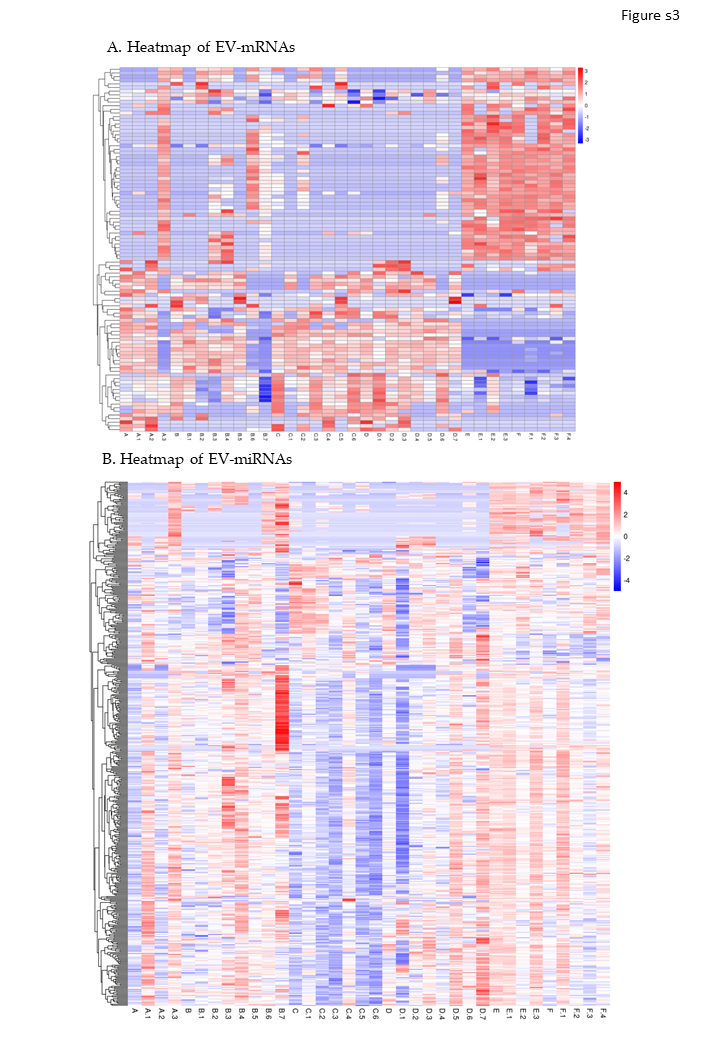

Supplement: Supplementary file 1 [file antioxidants-13-01047-s001.zip › Supplementary/Figure S3.TIF]

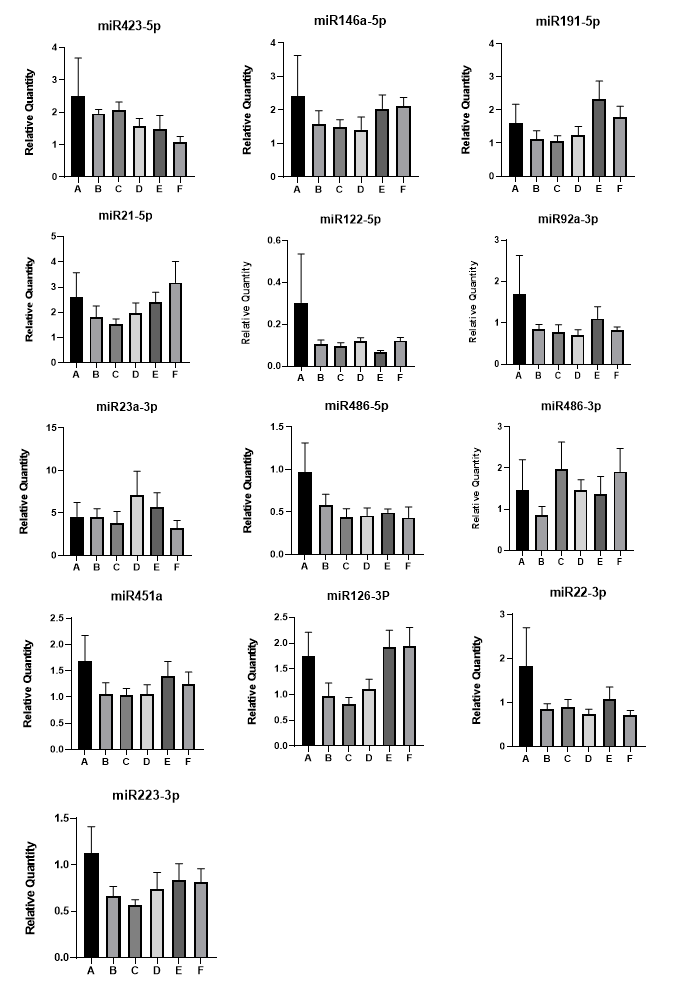

Supplement: Supplementary file 1 [file antioxidants-13-01047-s001.zip › Supplementary/Figure S4.TIF]

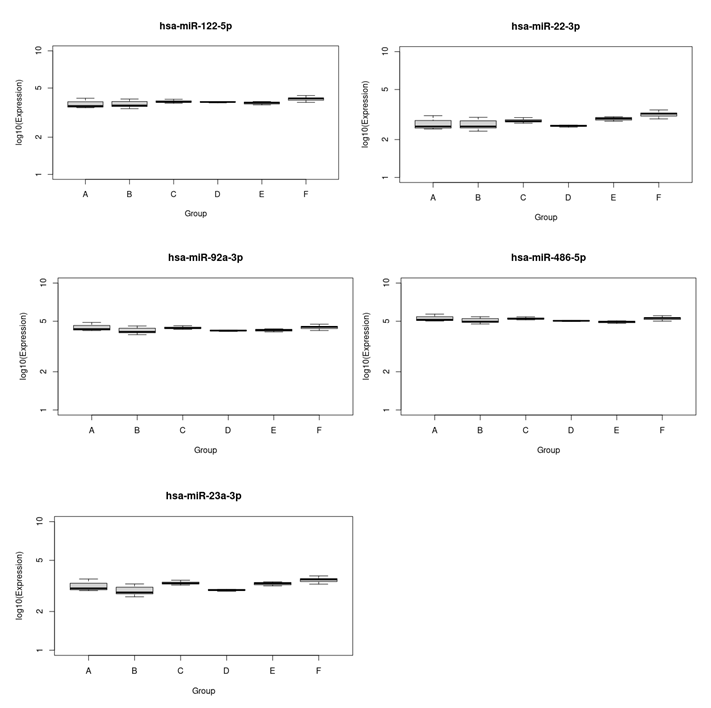

Supplement: Supplementary file 1 [file antioxidants-13-01047-s001.zip › Supplementary/Figure S5.TIF]
